# Childhood Socioeconomic Status and Adult Subjective Wellbeing: The Role of Hope and Sense of Control

**DOI:** 10.3389/fpsyg.2022.879132

**Published:** 2022-07-04

**Authors:** Li Wang, Fenglan Li, Keqiang Meng, Kelly Heber Dunning

**Affiliations:** ^1^College of Economics and Management, Huazhong Agricultural University, Wuhan, China; ^2^School of Forestry and Wildlife Sciences, Auburn University, Auburn, AL, United States; ^3^College of Marxism, Huazhong Agricultural University, Wuhan, China; ^4^School of Marxism, Shanghai Jiao Tong University, Shanghai, China

**Keywords:** childhood socioeconomic status, hope, sense of control, subjective wellbeing, mediation effects

## Abstract

The study investigates the unexplored link between childhood socioeconomic status and adult subjective wellbeing using data from a field survey of 568 rural residents from poor areas in China. This study focuses on exploring the relationship between childhood socioeconomic status, hope, sense of control, and adult subjective wellbeing using a structural equation model. Results indicated that hope and sense of control mediated the links between childhood socioeconomic status and adult subjective wellbeing, revealing that hope and sense of control may buffer the negative impacts of childhood poverty experiences on subjective wellbeing. The findings provide new insights into the impacts of childhood socioeconomic status on adult subjective wellbeing and expand the literature on key factors in adult subjective wellbeing.

## Introduction

An increasingly large body of study in social psychology revealed that childhood poverty is prospectively linked to adult outcomes including attainment-related outcomes (adult earnings and work hours) (Duncan et al., 2010, 2011), health outcomes (physical morbidity and mortality, psychological wellbeing) (Claussen, 2003; Howell and Howell, 2008; Cohen et al., 2010; Evans, 2016; Caria and Falco, 2018). Previous studies emphasized the impacts of childhood poverty on adult outcomes. However, it may be more important and valuable to tackle the issue that how to intervene to reduce the negative impacts of childhood poverty experiences on adult outcomes (e.g., subjective wellbeing). Particularly, how to weaken the negative impacts of lower childhood socioeconomic status on adult subjective wellbeing? One key to answer this question is finding more protective factors that may help ward off the negative impacts of lower childhood socioeconomic status on adult subjective wellbeing. Disappointedly, we still known little about the influence mechanism of childhood socioeconomic status on adult subjective wellbeing.

Hope is a positive psychological factor affecting subjective wellbeing (Diener and Biswas-Diener, 2002; Dittmar et al., 2014). Hope positively predicted subjective wellbeing (Cotton Bronk et al., 2009; Sariçam, 2015; Satici et al., 2020). Indeed, previous studies have highlighted the mediating role of hope (Cotton Bronk et al., 2009; Satici et al., 2020). For example, Chitchai et al. (2020) confirmed that hope for money mediated the relationship between socioeconomic status and happiness. Adult hope was based on the development of children’s brain and cognition (Glewwe et al., 2017), which is influenced by childhood socioeconomic status (Farah et al., 2006; Hair et al., 2015). Does childhood socioeconomic status affect adult subjective wellbeing through the mediating role of hope?

Sense of control is another potential mediating factor related to the relationship between childhood experiences and adult subjective wellbeing. Adult sense of control was closely related to the social class where they grew up (Kraus et al., 2012). Studies have emphasized the mediating role of sense of control in the relationship between discrimination experience and subjective wellbeing (Moradi and Hasan, 2004; Jang et al., 2008). And lower childhood socioeconomic status probably caused higher risks of discrimination experiences (Lang, 2011; Fuller-Rowell et al., 2012; Lee et al., 2018). The perceived experiences of prejudice and discrimination negatively affected their overall sense of control (Ruggiero and Taylor, 1995; Branscombe and Ellemers, 1998).

Therefore, this study focused on the mediating mechanism of childhood socioeconomic status and adult subjective wellbeing, aiming to explore the relationship between childhood socioeconomic status, hope, sense of control, and adult subjective wellbeing. Besides, we expected that hope and sense of control, as positive psychological quality, may help defend against the negative impacts of lower childhood socioeconomic status on adult subjective wellbeing. The results of this study may explain how childhood socioeconomic status affects adult subjective wellbeing from a psychological perspective. And the findings provided new empirical evidences and solutions for finding more mediating variables and reducing the negative impacts of childhood poverty experiences on adult subjective wellbeing.

To tackle this issue, we surveyed the rural residents in Jianshi County. Jianshi County, a key area of poverty intervention, is a typical poverty-stricken county in China. Over the long term, the mountainous area, poor transportation and poor water quality have resulted in a considerable number of poor people. In order to survive, many families become migrant workers for income, resulting in many left-behind children raised by grandparents or mother. According to a survey, there were 875 left-behind children among 1,917 children, and the left-behind rate reached 45.64% (Jv et al., 2015). Rural left-behind children with insufficient parents’ concern are often accompanied by risks of depression, perceived discrimination, parenting style, single-parent families (Wen and Lin, 2011; He et al., 2012; Wang et al., 2019). However, many adults in Jianshi County have experiences of childhood poverty and left-behind children.

The paper is structured as follows. Section “Literature Review and Theorization of Hypotheses” briefly reviews previous studies on the relationship between childhood socioeconomic status, hope, sense of control, and subjective wellbeing. Section “Aims and Hypotheses” introduces our hypotheses. The data and methods we used in the analysis are presented in Section “Data and Methodology.” Section “Results” presents the results of data analysis and reveals the relationships among the targeted variables. Section “Discussion and Conclusion” completes the paper with the conclusion and discussion.

## Literature Review and Theorization of Hypotheses

### Childhood Socioeconomic Status and Adult Subjective Wellbeing

Lower childhood socioeconomic status is an important indicator of childhood poverty. Several studies have confirmed that childhood poverty negatively affects adult subjective wellbeing (Oshio et al., 2009). Childhood poverty suggested higher risk of childhood adversity (Frederick and Goddard, 2007; Hughes and Tucker, 2018). Studies have demonstrated that childhood adversity experiences had substantial negative impacts on adult subjective wellbeing (Oshio et al., 2013). Lee et al. (2017) also found that childhood adversities were associated with poor adult mental health outcomes. Mwachofi et al. (2020) confirmed that adults with adverse childhood experiences including parents quarreling and beating each other during childhood are more likely to fall into depression and lower subjective wellbeing. In addition, Evidence suggested that early childhood poverty had detrimental impacts on adult education, career opportunities, earnings and work hours (Cohen et al., 2010; Duncan et al., 2010, 2011; Duncan and Magnuson, 2013). And higher social class (household income) was associated with greater happiness (Piff and Moskowitz, 2018). Income (Ferrer-i-Carbonell, 2005; Clark et al., 2008) and education (Cuñado and de Gracia, 2011; Kristoffersen, 2018; Tan et al., 2020) positively affected adult subjective wellbeing. Both Ferrer-i-Carbonell (2005) and Kristoffersen (2018) used an eleven-point numeric scale between 0 and 10 to measure subjective wellbeing (Cantril, 1965). Therefore, the first hypothesis is stated as follows:

**Hypothesis 1c**. Childhood socioeconomic status positively affects adult subjective wellbeing.

### Hope, Sense of Control, and Subjective Wellbeing

#### Hope and Subjective Wellbeing

Hope is a cognitive set and is defined as the agency and pathways to achieve desired goals (Snyder, 1995; Snyder et al., 2005). Hope represents an individual’s positive expectation for the future. Higher hope means that an individual has stronger desires to pursue future goals and a greater belief in their capacity to achieve them (Snyder et al., 1991, 1996). It is also a positive cognitive state or kind of coping strategy where one believes that things are going in the right direction and something is worth working or fighting for Havel (1990) and Donaldson et al. (2014). Normally, higher hope can lead to better outcomes in academics, athletics, physical health, mental health, and emotional adjustment (Snyder, 2002). Shorey et al. (2003) revealed that hope was closely related to adult mental health, and people with higher hope had less depression, less anxiety. Several studies found that hope positively predicted subjective wellbeing, although different scales were used to measure subjective wellbeing (Cotton Bronk et al., 2009; Sariçam, 2015; Satici et al., 2020). Cotton Bronk et al. (2009) used the Satisfaction with Life Scale (Diener et al., 1985) to assess the global cognitive judgments of satisfaction with one’s life. Sariçam (2015) and Satici et al. (2020) assessed subjective wellbeing by using the Subjective Happiness Scale (Lyubomirsky and Lepper, 1999). Specially, Chitchai et al. (2020) confirmed that hope for money positively affected happiness which was assessed with overall appreciation of one’s life as a whole (Veenhoven, 2012). Sometimes, hope can be seen as a kind of psychological protective factor associated with difficult conditions. Even under challenging life conditions, individuals with high hope have the strength to find alternative solutions and succeed (Satici et al., 2020). Therefore, the hypothesis is stated as follows:

**Hypothesis 2**. Hope positively affects adult subjective wellbeing.

#### Sense of Control and Subjective Wellbeing

Subjective wellbeing is an important positive aspect of mental health. A series of studies supported that sense of control is closely linked to mental health (Lachman and Weaver, 1998; Wolinsky et al., 2003; Moradi and Hasan, 2004; Kiecolt et al., 2009; Klama and Egan, 2011). Sense of control was defined as perceptions and beliefs about the ability to change the external environment and the future (Rodin, 1986; Burger, 1989). Lachman and Weaver (1998) found that greater sense of control was associated with higher life satisfaction and lower levels of depression. Empirical research by Moradi and Hasan (2004) confirmed that sense of control positively affected self-esteem and negatively affected psychological distress. According to Kiecolt et al. (2009), sense of control negatively affected psychological distress and any mental disorder, suggesting that sense of control tended to be monotonically related to positive mental health. Similarly, sense of control negatively affected anxiety and depression (Klama and Egan, 2011). Furthermore, losing control is one of the greatest fears of mankind (Astin and Shapiro, 1997). As an important factor affecting the life status of people throughout their lifespan (Rodin, 1986; Mirowsky, 1995), Greene and Britton (2015) found that sense of control positively predicted subjective wellbeing assessed by Subjective Happiness Scale (Lyubomirsky and Lepper, 1999). Therefore, the hypothesis is stated as follows:

**Hypothesis 3**. Sense of control positively affects adult subjective wellbeing.

### Childhood Socioeconomic Status, Hope, and Sense of Control

#### Childhood Socioeconomic Status and Hope

A few studies have demonstrated that adult hope was closely related to childhood hope (Kraftl, 2008; Bruner, 2017). Childhood hope can help individuals cope with childhood adversity (Munoz et al., 2020) and grow into adults with positive, optimistic character traits and hope for the future. Especially in poor families, it was crucial to build hope for children in early childhood stages (Glewwe et al., 2017). Meanwhile, childhood poverty inhibited the healthy development of children’s brain and cognitive including childhood hope (Pautler and Lewko, 1987; Farah et al., 2006; Hair et al., 2015), causing less hope during adulthood. In addition, Shorey et al. (2003) pointed out that adult hope was influenced by parenting styles directly and indirectly. Parenting styles positively affected adult hope and adults who grew up under the more positive and tolerant parenting styles (such as democracy) had higher hope (Kashdan et al., 2002). However, there existed many differences in parenting styles between poor and non-poor families (Magnuson and Duncan, 2002; Laplaca and Corlyon, 2015). Some studies found that lower childhood socioeconomic status was linked to negative parenting styles (Middlemiss, 2003; Hughes et al., 2005; Kaiser et al., 2017). Childhood family income and parental knowledge reflected by childhood socioeconomic status affected parenting styles (Goodman, 2007; September et al., 2015; Dix and Moed, 2019). Parents with less parenting knowledge adopted negative and extreme parenting styles including domineering and doting (Parks and Smeriglio, 1986; Shumow et al., 1998; Winter et al., 2011). This is not conducive to children’s psychological and cognitive development (Kaiser et al., 2017), leading to children with negative temperaments (Padilla and Ryan, 2019). Besides, Lower childhood socioeconomic status was detrimental to children development (Seccombe, 2000), predisposing them to significant increases in adverse childhood experiences (Slack et al., 2004; Steele et al., 2016). Simultaneously, Allender (2014) confirmed that adverse childhood experiences affect adult hope directly. Thus, the hypothesis is stated as follows:

**Hypothesis 1a**. Childhood socioeconomic status positively affects adult hope.

#### Childhood Socioeconomic Status and Sense of Control

Childhood socioeconomic status represents the social class in which an individual grows up. The research by Kraus et al. (2012) argued that individuals who grew up in high social classes had significantly higher sense of control than those grew up in lower social classes, revealing that adult sense of control was significantly affected by childhood socioeconomic status. A few studies suggested that poverty was strongly associated with perceived discrimination (Lang, 2011; Fuller-Rowell et al., 2012; Lee et al., 2018). A series of studies confirmed prejudice and discrimination experiences always accompanied by childhood poverty negatively affected sense of control (Ruggiero and Taylor, 1995; Branscombe and Ellemers, 1998; Moradi and Hasan, 2004; Jang et al., 2008). Conversely, a warm and safe childhood experience helped promote one’s sense of control (Greene and Britton, 2015). Moreover, lower childhood socioeconomic status directly inhibited the healthy development of children’s brain and cognition including sense of control (Farah et al., 2006; Cohen et al., 2010; Hair et al., 2015). According to a survey by Mittal and Griskevicius (2014), poor children feel significantly less sense of control than wealthy children, though their study was conducted with children rather than adults.

Therefore, the hypothesis is stated as follows:

**Hypothesis 1b**. Childhood socioeconomic status positively affects adult sense of control.

## Aims and Hypotheses

**FIGURE 1:**
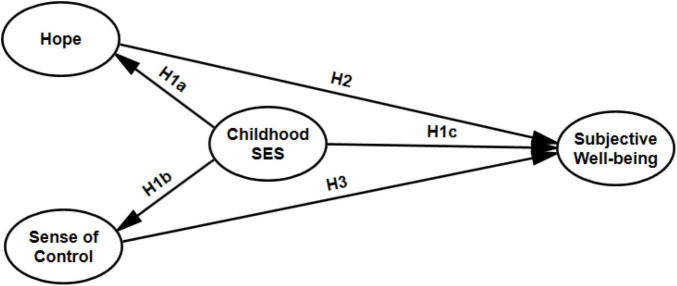
The proposed structural relationships between childhood socioeconomic status, hope, sense of control and subjective wellbeing.

In sum, the hypothesis is as follows: Childhood socioeconomic status is directly and indirectly through hope and sense of control related to adult subjective wellbeing. Based on the proposed hypothesis, we designed the model with mediators, which is described in Figure 1.

The purpose of this study is to expand on previous research by examining the relationship and influencing mechanisms between childhood socioeconomic status and adult subjective wellbeing. This study focuses on the relationship between childhood socioeconomic status, hope, sense of control, and subjective wellbeing. Particularly, we aim to reveal whether (and to what extent) hope and sense of control mediate the link between childhood socioeconomic status and adult subjective wellbeing.

## Data and Methodology

### Participants

568 rural residents (297 male, 271 female) participated in the survey. The mean age of participants was 48 (SD = 11.783), and the range was from 18 to 65. The participants’ education level was distributed as follows: 43.49% of the respondents had a primary education level (*n* = 247); 34.15% of the respondents had a junior high school education level (*n* = 194); 17.25% of the respondents had a high school education level (*n* = 98); and 5.11% of the respondents had a university education level (*n* = 29).

### Procedure

The research group, “Research on the Mental Health Promotion Strategy of Rural Residents under the Background of Targeted Poverty Alleviation” conducted a two-week questionnaire survey in Jianshi County, Hubei Province in July 2019. Jianshi County is a nationally recognized poverty-stricken county in China, consisting of 10 towns. To reduce the sample selection bias, the scope of this survey involves 8 towns and 19 villages in total, covering most of the area. Five villages in Huaping Town, the largest town in the county, were investigated, while 2 villages in each of all seven towns (Changliang Town, Gaoping Town, Hongyan Town, Guandian Town, Jingyang Town, Sanli Town, Maotian Town) were surveyed. 10–20 poor families and 10–20 non-poor families in each village were randomly selected to participate in the investigation. In addition, only one person (the head of household or his/her spouse) in each family was surveyed by two trained investigators through an interview-style questionnaire, where was conducted in the household’s home or in a common area of the workplace. Respondents who are literate and without functional limitation filled out the questionnaires themselves, and those who are illiterate or with functional limitation are asked to answer the questions through face-to-face interviews. A total of 600 questionnaires were handed out and 580 questionnaires were returned in the study. Questionnaire collection rate was 96.67%. Questionnaires with incorrect answers or incomplete information were removed. 568 valid questionnaires (270 poor samples, and 298 non-poor samples) were obtained among the 580 questionnaires and questionnaire-reclaiming efficiency was 94.67%. Adults with childhood poverty experiences are not certainly poor currently. Similarly, adults without childhood poverty experiences are not certainly out of poorness. Finally, 568 valid samples were conducted in the study.

### Instruments

All variables were measured by means of self-assessment. All the measurement instruments in this study directly used the translated Chinese version of the scales, which have been widely used in local Chinese studies.

#### Childhood Socioeconomic Status

Childhood socioeconomic status was assessed using the Chinese version of childhood socioeconomic status scale revised by Chinese scholar Yan et al. (2017), which was originally developed by Griskevicius et al. (2011a). Some studies have shown that Cronbach’s alpha is 0.805 (Yan et al., 2017) and 0.880 (Keye and Liuna, 2021) for the revised Chinese version of four-item scale, indicating high reliability and good measurement results. Childhood socioeconomic status was measured by participant recall during adulthood in this study. The measurement method was confirmed by many studies (Galobardes, 2004; Pollitt et al., 2005; Cohen et al., 2010; Griskevicius et al., 2011b, 2013; Belsky et al., 2012; Yan et al., 2017; Keye and Liuna, 2021), which provided strong support for this study. To assess childhood socioeconomic status, participants were made to respond to the following three statements with a nine-point scale from 1, “strongly disagree, “to 9, “strongly agree”:

1) “My family usually had enough money for things when I was growing up.”

2) “I grew up in a relatively wealthy neighborhood.”

3) “I felt relatively wealthy compared to the other kids in my school.”

4) “My parents had higher socioeconomic status during my childhood.”

A higher score means the respondent had a better life during childhood. The Cronbach’s alpha for the scale is 0.774. The mean value of childhood socioeconomic status was 2.861 (SD = 1.98), which was somewhat below the mid-score of the scale.

#### Hope

We measured hope using the Chinese version of State Hope Scale, which was originally developed by Snyder et al. (1996). Cronbach’s alpha is 0.830 (Zhihua, 2013) and 0.820 (Mengchao and Xiting, 2013) for the scale among Chinese adults, confirming good results for measuring hope among Chinese adults. The six items for the scale included the following:

1) “If I should find myself in a jam, I could think of many ways to get out of it.”

2) “There are lots of ways around any problem that I am facing now.”

3) “I can think of many ways to reach my current goals.”

4) “At present, I am energetically pursuing my goals.”

5) “Right now I see myself as being pretty successful.”

6) “At this time, I am meeting the goals that I have set for myself.”

An eight-point scale was used to evaluate the overall hope of the respondents. Respondents were made to answer each item according to the following choices: 1 (strongly disagree), 2 (mostly disagree), 3 (somewhat disagree), 4 (slightly disagree), 5 (slightly agree), 6 (somewhat agree), 7 (mostly agree), and 8 (strongly agree). A higher score represents stronger hope. Internal consistency for all the items yielded a Cronbach’s alpha of 0.805 for the scale. The mean value of hope was 4.966 (SD = 1.656), which was slightly above the mid-score of the scale.

#### Sense of Control

Sense of control was measured using Chinese version of sense of control scale translated by Jiahe (2007), which was originally developed by McConatha and Huba (1999). Cronbach’s alpha is 0.823 among college students with higher education (Xiaoyu, 2017) while Cronbach’s alpha is 0.6 among respondents with lower education (Jiahe, 2007), showing that the scale was suitable for measuring the sense of control among Chinese rural adults. The scale can assess the overall sense of control more comprehensively. It only includes three items and it is relatively simple and easy to understand for Chinese rural residents with lower education. The three items for the scale are as follows:

1) “I often feel that most situations are out of my control.”

2) “Usually, I feel that I have control over what is going on in my life.”

3) “Life is complicated, a person like me can’t understand what is going on.”

Respondents were asked to indicate their agreement on a five-point Likert scale, ranging from 1 (strongly disagree) to 5 (strongly agree). A higher score indicates a stronger sense of control. Internal consistency for all the items yielded a Cronbach’s alpha of 0.468 for the scale and the internal consistency seems to be approximately acceptable. The mean value of the sense of control was 3.054 (SD = 0.765), which was slightly above the mid-score of the scale.

#### Subjective Wellbeing

Adult subjective wellbeing was assessed using the Cantril Self-Anchoring Striving Scale developed by Cantril (1965), which is a schematic diagram of a ladder with eleven steps. The Cantril-ladder was used in many studies (Ferrer-i-Carbonell, 2005; Kahneman and Deaton, 2010; Glatzer and Gulyas, 2014; Kristoffersen, 2018) and is still used in the Gallup World Poll (Harter and Gurley, 2008). It is a measure of the cognitive aspect of subjective wellbeing which represents an overall assessment of life. The ladder scale can assess subjective wellbeing more comprehensively and it is a non-verbal scale that applied to different cultures and regions well, especially, it is suitable for surveying rural residents with low education. The graphical measurement instrument makes the scale avoid depending on a specific language environment, especially in rural China where there are many dialects. Participants were made to select the current position ranging from 0 (worst possible life) to 10 (best possible life) according to their own criteria. The higher score, the higher subjective wellbeing. The mean value of subjective wellbeing was 5.511 (SD = 2.174), which was slightly above the mid-score of the scale.

#### Control Variables

Demographic variables such as gender, age, education and average household income were also controlled in the model. Males generally have lower subjective wellbeing than females (Knight et al., 2009). According to Easterlin (1995), high-income groups have a higher level of subjective wellbeing compared to low-income groups.

### Statistical Analysis

Statistical analysis of data in the study was carried out through STATA14.0 and AMOS24.0. Firstly, we examined common method biases, the reliability and validity of measurement scales. And descriptive statistics and correlations of variables were confirmed. Secondly, we tested the theoretical model in Figure 1 using structural equation modeling (SEM) and evaluated goodness of fit of model. Thirdly, we conducted the mediation testing of hope and sense of control using the bootstrapping method in the case of the 1000 samples taken *via* AMOS24.0. In addition, we compared the impacts of childhood socioeconomic status on subjective wellbeing between the direct model (model 1: without mediator) and the mediation model (model 2: with mediator).

## Results

### Common Method Biases Test

Harman’s single-factor test was used to examine the problem of common method biases. The results showed that there are 4 factors with eigenvalues greater than 1. And the first factor explained 29.44% of the total variance, which is less than the critical value of 40% (Podsakoff et al., 2003), indicating that there is no obvious common method biases problem in this study.

### Reliability and Validity Test Results of Scales

Before further investigation, we firstly tested the reliability and validity of the scales. We calculated the reliability index (Cronbach’s α, construct reliability, factor loading of each factor) and validity index [average variance extracted (AVE) and $\sqrt{A\,V\,E}$]. Tables 1, 2 show reliability and validity test results, the descriptive statistics, and Pearson’s correlations. Internal consistency seems to be approximately acceptable although the reliability results suggests that future research should pay more attention to the revision of the sense of control scale. In addition, AVE of all variables are greater than 0.3. Moreover, $\sqrt{A\,V\,E}$ are greater than correlation coefficients between all variables, indicating that the measured instruments has good convergence validity and discriminative validity according to Fornell and Larcker (1981) and Tabachnick et al. (2007).

**TABLE 1:** Reliability and convergence validity.

Dimensions	Item	Mean	SD	Factor loading	Cronbach’s α	CR	AVE
Childhood SES	CSES01	2.215	2.173	0.611	0.774	0.784	0.477
	CSES02	2.526	2.515	0.667			
	CSES03	3.387	2.697	0.713			
	CSES04	3.315	2.825	0.763			
Hope	Hope01	5.456	2.325	0.605	0.805	0.793	0.406
	Hope02	5.558	2.354	0.505			
	Hope03	4.845	2.288	0.771			
	Hope04	4.520	2.275	0.400			
	Hope05	4.642	2.339	0.873			
	Hope06	4.773	2.379	0.544			
Sense of Control	Control01	2.942	1.227	0.767	0.468	0.528	0.300
	Control02	3.588	0.990	0.395			
	Control03	2.632	1.066	0.376			

**TABLE 2:** Descriptive statistics, Pearson’s correlations and the discriminatory validity.

	M	SD	1	2	3
1. Childhood SES	2.861	1.980	(0.691)		
2. Hope	4.966	1.656	0.323***	(0.637)	
3. Sense of control	3.054	0.765	0.150***	0.385***	(0.548)
4. Subjective wellbeing	5.511	2.174	0.190***	0.269***	0.297***

N = 568. Square root value of average variance extracted is in parenthesis. ***p < 0.001.

### Goodness of Fit Test of Structural Equation Model

To be ensure the degree of fitness between the data and the structural equation model, we conducted a goodness-of-fit test on the model through the AMOS24.0 software. Table 3 shows the goodness of fit test results of structural equation model. According to the criteria proposed by Lance et al. (2007) and Kim et al. (2009), the results showed that the model represented a good fit for the data, and thus can be used in the study [χ^2^/*df* = 1.383; RMSEA = 0.026; GFI = 0.976; AGFI = 0.951; IFI = 0.985; CFI = 0.984; TLI = 0.971; 90 percent confidence interval for RMSEA = (0.015; 0.036)].

**TABLE 3:** Test of Goodness of fit index of the model.

Goodness of fit index	χ2/df	RMSEA	GFI	AGFI	IFI	CFI	TLI
Test value	1.383	0.026	0.976	0.951	0.985	0.984	0.971
Reference value	< 3	< 0.080	> 0.900	> 0.900	> 0.900	> 0.900	> 0.900

### Mediation Analysis

We conducted the mediation testing of hope and sense of control using the bootstrapping method in the case of the 1000 samples taken *via* AMOS24.0. Table 4 shows the results of the mediation testing using the bias-corrected percentile method and percentile method. It is found that the lower and upper bound values of the indirect effects of childhood socioeconomic status on subjective wellbeing is 0.078 and 0.268, respectively by using the bias-corrected percentile method. The confidence interval (0.078, 0.268) does not include 0, suggesting that hope and sense of control play a mediating role in the relationship between childhood socioeconomic status and subjective wellbeing. Furthermore, as shown in Table 4, the indirect effects of mediated pathways between childhood SES and subjective wellbeing are 0.153.

**TABLE 4:** Testing results of the mediation effects using the Bias-corrected percentile method and percentile method.

Path relationship	Effect			SE	95% Confidence interval			
			
					Bias-corrected percentile		Percentile	
						
	Total	Direct	Indirect		Lower	Upper	Lower	Upper
CSES → Hope + Sense of Control→ Subjective Wellbeing	0.324	0.171	0.153	0.047	0.078	0.268	0.072	0.259

*N = 568, bootstrap sample size = 1000.*

Table 5 illustrates the direct effects results of mediation model. As shown in Table 5, childhood socioeconomic status has significant positive impacts on hope (β = 0.342, *p* < 0.001) and hypothesis H1a was confirmed. Similarly, childhood socioeconomic status has significant positive impacts on sense of control (β = 0.028, *p* < 0.05) and hypothesis H1b was confirmed. Childhood socioeconomic status has significant positive impacts on subjective wellbeing (β = 0.171, *p* < 0.05) and hypothesis H1c was confirmed. Hope has significant positive impacts on subjective wellbeing (β = 0.301, *p* < 0.001), confirming hypothesis H2. Sense of control has significant positive impacts on subjective wellbeing (β = 1.794, *p* < 0.001), confirming hypothesis H3.

**TABLE 5:** The direct effects results of mediation model.

Hypothesis	Direct effects	Unstd. estimate	S.E.	Result
H1a	CSES → Hope	0.342***	0.063	Yes
H1b	CSES → Sense of Control	0.028*	0.013	Yes
H1c	CSES → Subjective Wellbeing	0.171*	0.082	Yes
H2	Hope → Subjective Wellbeing	0.301***	0.083	Yes
H3	Sense of Control → Subjective Wellbeing	1.794***	0.420	Yes

N = 568. ***p<0.001, *p<0.01.

**FIGURE 2:**
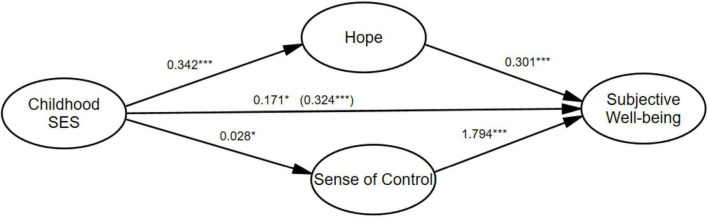
Unstandardized estimated path coefficients of the structural equation model. *N* = 568. Model 1: Childhood SES → Subjective Wellbeing (without mediator). Model 2: Childhood SES → Hope + Sense of Control → Subjective Wellbeing. The numbers represent the beta coefficients for Model 2. The beta coefficients for Model 1 are in parentheses. ***p < 0.001, *p < 0.01.

We compared the impacts of childhood socioeconomic status on subjective wellbeing when there is hope and sense of control and when there is no hope and sense of control. Figure 2 shows the path coefficient results for the direct model (model 1: without mediators) and the mediation model (model 2: with mediators) between childhood socioeconomic status and subjective wellbeing. As shown in the results, the direct effects of childhood socioeconomic status on subjective wellbeing in the model without mediators are 0.324 (*p* < 0.001). However, the direct effects of childhood socioeconomic status on subjective wellbeing in the model with mediators are 0.171 (*p* < 0.05). Interestingly, the direct effects of childhood socioeconomic status on subjective wellbeing are significantly smaller when the mediators are taken into the model. The results demonstrated hope and sense of control mediated the links between childhood socioeconomic status and adult subjective wellbeing. What is more important, it revealed that hope and sense of control may buffer the impacts of childhood socioeconomic status on subjective wellbeing.

## Discussion and Conclusion

Our study findings supported Hypothesis 1a, Hypothesis 1b, Hypothesis 1c, Hypothesis 2, and Hypothesis 3. Mediation analysis results indicated that childhood socioeconomic status was directly and indirectly through hope and sense of control related to adult subjective wellbeing.

One of our findings was that childhood socioeconomic status had direct and positive impacts on adult subjective wellbeing, implying that the negative impacts of poverty on children may extend throughout adulthood. This result was supported by previous studies (Duncan and Magnuson, 2013; McCarty, 2016). Adults who grew up in poor families tend to have lower educational attainment, face higher poverty risks, and assess themselves as being less happy (Oshio et al., 2009). The finding in this study was consistent with Oshio et al. (2009), demonstrating that the impacts of childhood socioeconomic status on adult subjective wellbeing is more or less direct. Research by Cohen et al. (2010) also confirmed that adults with higher childhood socioeconomic status were more likely to have positive social emotions.

A possible explanation is that lower childhood socioeconomic status was often accompanied by adverse childhood experiences including domestic violence (Gelles, 1992; Brandwein, 2007), discrimination (Branscombe and Ellemers, 1998; Lang, 2011; Fuller-Rowell et al., 2012; Lee et al., 2018), single-parent households (Jonson-Reid et al., 2013), lower parental education and poor parenting (Hughes et al., 2005; Laplaca and Corlyon, 2015; September et al., 2015), poorer educational resources and facilities (Evans and Kantrowitz, 2002), poorer housing quality and smaller house (Gİtmez and Morcöl, 1994; Evans, 2006). These social stressors are bound to cause many negative effects on children’s mental health. Moreover, the negative impacts of low socioeconomic status on children may accumulate over time or lie dormant for years. The long-term negative impacts were revealed during adulthood and persist throughout life (McLeod and Shanahan, 1996; Brooks-Gunn and Duncan, 1997; Foster and Furstenberg, 1999; Ratcliffe and McKernan, 2010; McCarty, 2016). For example, adults with adverse childhood experiences including parents quarreling and beating each other during childhood are more likely to fall into depression (Mwachofi et al., 2020). Besides, to a large extent, adult emotional responses, behaviors, and decisions are determined by childhood socioeconomic status (Griskevicius et al., 2013). Adults who grew up in the context of higher childhood socioeconomic status were more rational and more likely to make right decisions. Consequences of right decisions and behaviors are conducive to subjective wellbeing (Mittal and Griskevicius, 2014).

Another important result in our study indicated that hope and sense of control mediated the links between childhood socioeconomic status and adult subjective wellbeing. As shown in the results, the direct effects of childhood socioeconomic status on subjective wellbeing in the model without mediators are 0.324. However, the direct effects of childhood socioeconomic status on subjective wellbeing in the model with mediators are 0.171. The direct effects of childhood socioeconomic status on subjective wellbeing are significantly smaller when the mediators are taken into the model, revealing that hope and sense of control may buffer the negative impacts of childhood poverty experiences on subjective wellbeing. Our research not only explained how childhood socioeconomic status affected adult subjective wellbeing from a cognitive perspective, but also confirmed the points by Cohen et al. (2010) and Chitchai et al. (2020) that childhood socioeconomic status was mainly linked to adult subjective wellbeing through internal psychological mechanisms. Our results are consistent with Satici et al. (2020), demonstrating that hope is positively related to subjective wellbeing. The main reason may be that adults with higher expectations were good at balancing multiple social roles (work, marriage, family) and having more intimate and harmonious relationships with colleagues, partners, and children, resulting in higher happiness (Kashdan et al., 2002). Our study also confirms that childhood socioeconomic status is positively related to sense of control. As explained by Zhou et al. (2009), adults who grew up in higher social classes had higher sense of security, more self-confidence, stronger problem-solving skills, causing higher sense of control.

The most important contribution of this research is that our study confirms the buffering effects of mediating variables (hope and sense of control) on the negative impacts of lower childhood socioeconomic status on adult subjective wellbeing. The paper highlights the importance of hope and sense of control in the relationship between childhood socioeconomic status and subjective wellbeing. Even if adults grew up in poverty during childhood, we can reduce the cost of childhood poverty on adult subjective wellbeing by intervening in hope and sense of control. In fact, we can also see that a large number of adults suffered from childhood poverty in real life still have higher subjective wellbeing. The study also implies that future research should focus on exploring the various mediating variables between childhood socioeconomic status and adult subjective wellbeing, and considering how to intervene these mediator variables to reduce the negative impacts of childhood poverty experiences on adult subjective wellbeing.

Another important contribution of this study is that our study explores the internal mechanism of how childhood socioeconomic status affects adult subjective wellbeing from the perspective of psychology. Most studies considered how to improve people’s wellbeing through outcomes including education and income while ignoring the role of the individual’s intrinsic cognitive function. Our findings suggests that hope and sense of control are key factors to consider when exploring the impacts of childhood poverty on adult subjective wellbeing. This paper provides new insights into the impacts of childhood socioeconomic status on adult subjective wellbeing and expand the literature on key elements of adult subjective wellbeing. In particular, based on micro-survey data from poor rural areas in China, this study provides evidences that childhood socioeconomic status affects adult subjective wellbeing in non-western cultural contexts.

In addition, this study has some practical implications. This study guides us to pay more attention to children with lower socioeconomic status, in particular, to emphasize the impacts of socioeconomic status on their hope and sense of control. As pointed out by McCarty (2016), hope of resilience through policies and programs were offered to reduce child poverty and mitigate its damages (McCarty, 2016). Moreover, Adult hope was closely related to childhood hope (Kraftl, 2008; Bruner, 2017). Especially for families with lower socioeconomic status, it was crucial to build hope for them in early childhood (Glewwe et al., 2017). Therefore, in addition to implementing financial assistance to ensure children and adolescents’ basic living security, it also emphasizes that their hope and sense of control can be fostered through early family intervention, school education, and third-party social support in the process of practical intervention. For example, encourage self-presentation and provide more opportunities for self-expression, learning and communication with the outside world.

Future research should focus on improving three aspects: Firstly, it will be more accurately to reflect the impacts of childhood socioeconomic status *via* conducting a longitudinal study, as well as the effects of childhood socioeconomic status on individuals’ subjective wellbeing at different time throughout the lifespan. Secondly, further studies are necessary to focus on exploring more mediating variables between childhood socioeconomic status and adult subjective wellbeing, and considering how to intervene these mediators to reduce the negative impacts of childhood poverty experiences on adult subjective wellbeing. Thirdly, future research in this field should focus on the role of family education on the relationship between childhood socioeconomic status and hope and sense of control.

## Limitations

Firstly, this study focuses on the subjective wellbeing of residents in rural, poverty-stricken areas in China, excluding rural residents in non-poor areas. Future research can expand the range of investigation for comparative analysis. Secondly, adults were asked to recall their SES during childhood in our study and these recollections may be biased. This suggests that future studies can improve this study by test the study’s hypothesis using longitudinal data that assess pathways connecting childhood SES, mediators, adult wellbeing across multiple time points in the life span. Thirdly, the Cronbach’s alpha value of sense of control scale in this study is a bit low, suggesting that further research should pay attention to the development and revision of the sense of control scale for rural residents. Lastly, the direction between variables can be other than we assumed and we did not verify that. For example, childhood socioeconomic status can be indirectly related to hope and sense of control *via* adult subjective wellbeing.

## Data Availability Statement

The original contributions presented in this study are included in the article/Supplementary Material, further inquiries can be directed to the corresponding author.

## Author Contributions

FL designed the work and the field survey. LW, KM, and FL collected the data. LW analyzed the data and experiment’s results and wrote the manuscript. KD modified grammar and expressions of the manuscript. All authors contributed to the article and approved the submitted version.

## Conflict of Interest

The authors declare that the research was conducted in the absence of any commercial or financial relationships that could be construed as a potential conflict of interest.

## Publisher’s Note

All claims expressed in this article are solely those of the authors and do not necessarily represent those of their affiliated organizations, or those of the publisher, the editors and the reviewers. Any product that may be evaluated in this article, or claim that may be made by its manufacturer, is not guaranteed or endorsed by the publisher.
